# Menstrual Cycle Length and Patterns in a Global Cohort of Women Using a Mobile Phone App: Retrospective Cohort Study

**DOI:** 10.2196/17109

**Published:** 2020-06-24

**Authors:** Jessica A Grieger, Robert J Norman

**Affiliations:** 1 Robinson Research Institute University of Adelaide Adelaide Australia; 2 Adelaide Medical School University of Adelaide Adelaide Australia; 3 Fertility SA Adelaide Australia

**Keywords:** body mass index, follicular phase, fertility, luteal phase, menstrual cycle, mobile application, internet, ovulation, mobile phone

## Abstract

**Background:**

There is increasing information characterizing menstrual cycle length in women, but less information is available on the potential differences across lifestyle variables.

**Objective:**

This study aimed to describe differences in menstrual cycle length, variability, and menstrual phase across women of different ages and BMI among a global cohort of Flo app users. We have also reported on demographic and lifestyle characteristics across median cycle lengths.

**Methods:**

The analysis was run based on the aggregated anonymized dataset from a menstrual cycle tracker and ovulation calendar that covers all phases of the reproductive cycle. Self-reported information is documented, including demographics, menstrual flow and cycle length, ovulation information, and reproductive health and diseases. Data from women aged ≥18 years and who had logged at least three cycles (ie, 2 completed cycles and 1 current cycle) in the Flo app were included (1,579,819 women).

**Results:**

Of the 1.5 million users, approximately half (638,683/1,579,819, 40.42%) were aged between 18 and 24 years. Just over half of those reporting BMIs were in the normal range (18.5-24.9 kg/m^2^; 202,420/356,598, 56.76%) and one-third were overweight or obese (>25 kg/m^2^; 120,983/356,598, 33.93%). A total of 16.32% (257,889/1,579,819) of women had a 28-day median cycle length. There was a higher percentage of women aged ≥40 years who had a 27-day median cycle length than those aged between 18 and 24 years (22,294/120,612, 18.48% vs 60,870/637,601, 9.55%), but a lower percentage with a 29-day median cycle length (10,572/120,612, 8.77% vs 79,626/637,601, 12.49%). There were a higher number of cycles with short luteal phases in younger women, whereas women aged ≥40 years had a higher number of cycles with longer luteal phases. Median menstrual cycle length and the length of the follicular and luteal phases were not remarkably different with increasing BMI, except for the heaviest women at a BMI of ≥50 kg/m^2^.

**Conclusions:**

On a global scale, we have provided extensive evidence on the characteristics of women and their menstrual cycle length and patterns across different age and BMI groups. This information is necessary to support updates of current clinical guidelines around menstrual cycle length and patterns for clinical use in fertility programs.

## Introduction

### Background

For more than 30 years, the notion has been that the average woman is potentially fertile between days 10 and 17 of her 28-day menstrual cycle. However, this assumes that ovulation occurs exactly 14 days before the onset of the next menstrual period and that the fertile window extends before and after ovulation [1]. Data on aggregated cycles show mean cycle lengths of 28 to 29 days [2,3] and 29 to 30 days [4-7]. Other studies reporting on the number of women and their cycle length have demonstrated that 77% of women in the United States and Canada have a cycle length of 25 to 31 days [5]; 65% of rural Chinese women have a cycle length of 27 to 29 days [8]; and 84% of Australian women have a cycle length of 26 to 34 days [9]. Few studies also reported on the estimated day of ovulation, demonstrating an estimated day of ovulation at day 14 in only 3% to 10% of cycles [10] and at day 15 in 12% to 16% cycles [10].

Landmark longitudinal [7,11] and cross-sectional [5] studies on menstrual cycle variability in the late 1960s and 1970s demonstrated that the majority of women had a cycle length between 15 and 45 days [5,7,11], with mean cycle lengths decreasing with increasing age. Subsequent studies have reported similar findings with a shorter cycle length and less variability with increasing age [3,12,13]. Studies among Western populations have shown that longer menstrual cycles were associated with a higher BMI [14], increased parity [14], and recent use of oral contraceptives [15], whereas shorter menstrual cycles were associated with smoking [14,16], heavy caffeine intake [17], and alcohol consumption [18]. There is much less information on other lifestyle and behavioral factors; however, women reporting depression, higher perceived stress levels, and high levels of physical activity were associated with having irregular periods, anovulation, and heavier menstrual bleeding [19-22], highlighting the impact that a range of modifiable factors may have on future fertility. Additionally, although early studies with small sample sizes show measures of obesity and body composition associated with menstrual cycle irregularities [23-26], the specific impact of BMI has not been clearly determined. This is important given the global increase in BMI, of which the rise in obesity prevalence has been most prominent in women of reproductive age [27].

For the past 10 years, hundreds of smartphone apps have been developed for women to track their menstrual cycles, ovulation and fertile days, and health information [4,28-32]. A calendar-based method app used with 949 women indicated a mean 28-day cycle (range 17-35 days), with the most likely day of ovulation being day 16 [33], and in 45,360 women using the *Ovia Fertility* app, the mean cycle length was 30.4 (SD 4.6) days [34]. In over 600,000 cycles collected using the *Natural Cycles* app, the mean cycle length was 29.3 (SD 5.2) days [4], and in 225,596 cycles*,* the mean cycle length was 29.6 (SD 5.4) days [35]. Despite this surge in population data of menstrual cycle length, information on *individual women* is lacking rather than aggregates of cycle length, and it remains unclear how many women actually have an *average* 28-day cycle. Furthermore, the characteristics of women according to cycle length and lifestyle are limited.

### Objectives

This study aimed to describe differences in menstrual cycle length, variability, and menstrual phase across women of different ages and BMIs. We also reported on demographic and lifestyle characteristics across the median cycle length. The study analyses will provide extensive worldwide evidence on the characteristics of menstrual cycle length and patterns among a global cohort of Flo app users. This information is necessary to support recommendations within current obstetric clinical guidelines around menstrual cycle length and patterns for clinical use in fertility programs.

## Methods

### Data Collection

The data were collected through the mobile period tracking and ovulation calendar called Flo, which was launched in October 2015. Users of the Flo app are found in over 200 countries, but the vast majority of them are located in Europe and North America. Flo’s privacy policy and terms of use permit the use of aggregated and anonymized data for research purposes. Data collection for this study started in December 2018 and extracted from 2 million users aged ≥18 years. Women were not included if they had logged a pregnancy, oral contraceptive reminders, and had recorded less than 3 menstrual cycles, which was the minimum number needed to obtain median cycle data. This resulted in a cohort of 1,579,819 women.

### App Content

The participants' ages were collected through the mandatory sign-up questionnaire, and BMI was calculated for women who reported their weight (the latest input was used) and height. Additional information was collected from the user in 2 ways: (1) by tracking the symptoms that users log themselves and (2) by survey answer questions. For example, the user can log information on cycle length, period length and intensity, and days of the luteal phase. The survey information that users can report includes a range of questions regarding diet, lifestyle (general well-being, sleep, sexual activity, physical activity, stress, alcohol), menstrual cycle symptoms, and reproductive health information. Manually logged information reported in the current analyses included information on weight (in kilogram, logged manually), menstrual flow (light, medium, heavy), smoking status (regularly, sometimes, do not smoke), alcohol consumption (≥3 times a week, 1-2 times a week, <2-3 times a month, none), frequency of high stress (≥3 times a week, 1-2 times a week, <3 times a month, none), frequency of physical activity (≥3 times a week, 1-2 times a week, <3 times a month, none), relationship status (married or stable partner, single, no partner), months trying to conceive (<1 month, 1-3 months, 3-6 months, >6 months to 1 year, >1 year), number of children (0, 1, or 2, ≥3), and any reproductive disorders (yes, no, I don't know).

### Menstrual Cycle Characteristics

Women manually logged information about their menstrual cycles (days of menstruation), including the intensity of menstrual flow (light, medium, heavy). The start and end dates of menstrual cycles were defined by the logged first day of menstruation. To estimate mean and median cycle lengths, we computed intrawomen inputs and then calculated the mean and median for population groups.

*Menstruation flow* calculation was completed for every cycle for each woman. If the length of a period was 1 or 2 days, it was calculated as low, but for a length of ≥3 days with more than half of the days logged with the intensity, it was calculated as light (L)=1, medium (M)=2, heavy (H)=3, none=no data. These numbers were arbitrary, as there is no measure of the volume of blood loss during menstruation. The average was computed for all bleeding days per cycle (eg, bleeding days: LMHHML=1+2+3+3+2+1=12 divided by 6=2=M). If the average was <1.5, it was recorded as light. If the average was ≥1.5 to <2.4, it was recorded as medium, and if the average was ≥2.4, it was recorded as heavy flow. In the case of ≥3 days logged and <50% days with reported intensities, it was considered as not tracked. Calculations were performed for all cycles that were logged by each woman.

*Ovulation* was recorded in women who reported a positive luteinizing hormone (LH) test, signifying the end of the follicular phase, and the next day estimated ovulation. In the case of multiple results logged, the last one was used. The length of the follicular phase was estimated from the first day of the menstrual cycle up to and excluding the day of ovulation. The length of the luteal phase was defined as the period from the day of ovulation up to and excluding the first day of menstruation.

*The cycle length variation* was calculated as the expected value of the mean square deviation, that is, var(x)=E[(x-mu)2], where E[.] is expectation and mu=E[x].

## Results

### Participant Characteristics

The data from over 1.5 million nonpregnant women who were not using oral contraceptives and who had logged at least 3 cycles (N=1,579,819) were included in the study (Table 1). The number of women with 2 completed and 1 current cycle was 123,245, with 3 to 10 cycles (n=819,933), 11 to 20 cycles (n=450,096), 21 to 30 cycles (n=154,536), and ≥31 cycles (n=32,009). Overall, the women were young, did not smoke, and rarely consumed alcohol but were mostly sedentary. That is, approximately half (638,683/1,579,819, 40.42%) of the users were aged 18 to 24 years. Only 22.57% (356,598/1,579,819) of the women reported their height and weight (to determine BMI); over half had a BMI in the normal range of 18.5 to 24.9 kg/m^2^ (202,420/356,598, 56.76%) and one-third (120,983/356,598, 33.93%) were overweight or obese. About half of the total population reported their smoking, alcohol, and physical activity habits, of which 25.73% (192,118/746,396) smoked, 25.10% (186,378/742,615) consumed alcohol ≥1 time per week, and 45.36% (363,314/800,870) did not engage in physical activity.

**Table 1:** Description of the study population.

Characteristics		Values, n (%)
**Age (years; n=1,579,819)**		
	18-24	638,683 (40.43)
	25-29	383,179 (24.25)
	30-34	275,039 (17.41)
	35-39	162,156 (10.26)
	40-55	120,762 (7.64)
**BMI (kg/m^2^; n=356,598)**		
	≤18.4	33,195 (9.31)
	18.5-24.9	202,420 (56.76)
	25.0-29.9	70,707 (19.83)
	30.0-34.9	30,144 (8.45)
	35.0-50.0	19,540 (5.48)
	≥50+	592 (0.17)
**Menstruation flow** (**n=197,579)**		
	Light	44,171 (22.36)
	Medium	148,236 (75.03)
	Heavy	5172 (2.62)
**Smoking status (n=746,369)**		
	Regularly	104,612 (14.01)
	Sometimes	87,506 (11.72)
	Do not smoke	554,278 (74.26)
**How often do you drink alcohol? (n=742,615)**		
	≥3 times a week	42,115 (5.67)
	1-2 times a week	144,263 (19.42)
	<2-3 times a month	310,597 (41.82)
	None	245,640 (33.08)
**Frequency of high stress** **(n=768,571)**		
	≥3 times a week	252,894 (32.90)
	1-2 times a week	248,630 (32.35)
	<3 times a month	218,490 (28.43)
	None	48,557 (6.32)
**Frequency of physical activity** **(n=800,870)**		
	≥3 times a week	140,287 (17.52)
	1-2 times a week	176,124 (22.00)
	<3 times a month	121,145 (15.13)
	None	363,314 (45.36)
**Relationship status** **(n=748,612)**		
	Married or stable partner	531,360 (70.98)
	Single	73,194 (9.78)
	No partner	144,058 (19.24)
**Trying to conceive** **(n=137,973)**		
	<1 month	36,510 (26.46)
	1-3 months	27,383 (19.85)
	3-6 months	17,108 (12.40)
	>6 months to 1 year	15,088 (10.94)
	>1 year	41,884 (30.36)
**Number of children** **(n=709,037)**		
	0	501,976 (70.80)
	1 or 2	178,303 (25.15)
	≥3	28,758 (4.06)
**Any reproductive disorders (n=687,290)**		
	Yes	76,722 (11.16)
	No	443,597 (64.54)
	I don’t know	166,971 (24.29)

### Menstrual Cycle Length and Characteristics

In all, 91.13% (1,439,613/1,579,819) of women had a median cycle length of 21 to 35 days, whereas 89.04% (1,406,643/1,579,819) had an average cycle length of 21 to 35 days. A total of 0.17% (2614/1,579,819) had a short cycle length (<21 days), and 8.60% (135,824/1,579,819) had a long cycle length (>35 days). The percentage of women with a median 27-day, 28-day, and 29-day cycle length was 12.05% (190,373/1,579,819), 16.32% (257,889/1,579,819), and 12.11% (191,351/1,579,819), respectively. Table 2 describes the demographic and lifestyle characteristics of the women, split by median cycle length. A higher percentage of women with short cycles reported having high stress ≥3 times/week (448/1117, 40.10%) and performing no physical activity (622/1158, 53.71%) compared with women with normal and long cycles. A higher percentage of women with short cycles had a lighter menstrual flow (240/476, 50.4%) compared with women with normal (37,469/172,025, 21.78%) and long cycles (6462/25,078, 25.77%). Consumption of alcohol or smoking status did not appear to influence menstrual cycle length (Table 2).

**Figure 1:**
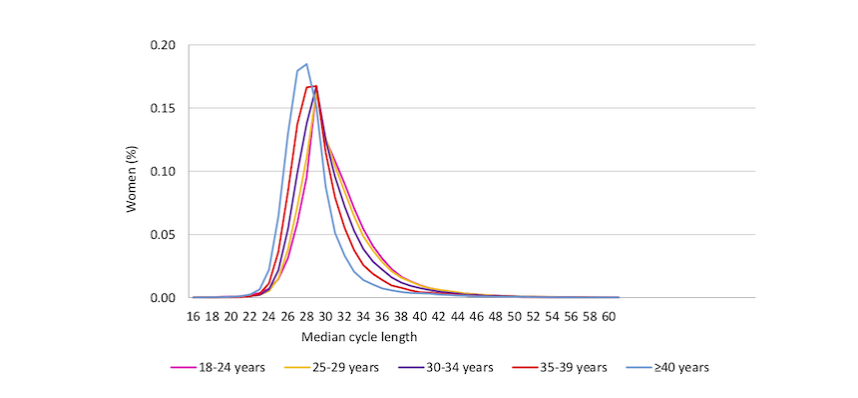
Median cycle length distribution by age groups.

**Figure 2:**
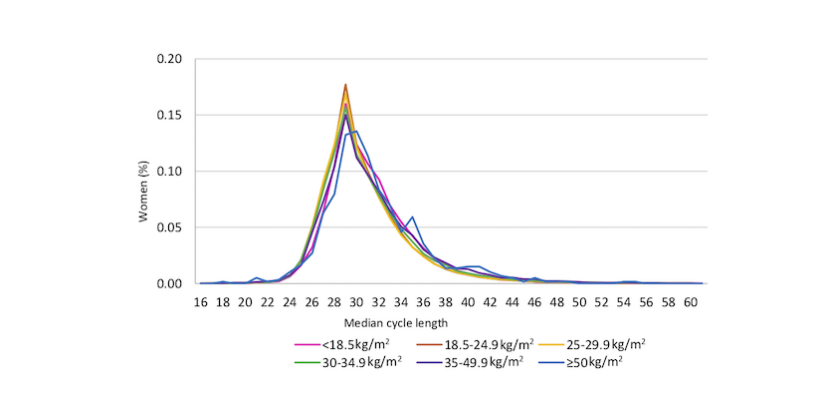
Median cycle length distribution by BMI categories.

Figures 1 and 2 show the median cycle length distribution by age and BMI, respectively. In women aged 35 to 39 years and ≥40 years, more than 16% had a median cycle length of 27 days, compared with 9.6% of women aged 18 to 24 years. Comparatively, <8% of women aged 35 to 39 years and ≥40 years had a median 30-day cycle length compared with 10.8% of women aged 18 to 24 years. For BMI <50 kg/m^2^, the highest percentage of women had a 28-day median cycle (15%-18%) and tended to track similarly across BMIs for shorter and longer day cycles (Figure 2). There were 592 women (0.17% of all women) with a BMI of ≥50 kg/m^2^; the highest percentage had a median 29-day cycle (n=80, 13.6%). A higher percentage of women with a BMI of 35 to 50 kg/m^2^ (11.6%) and >50 kg/m^2^ (11.7%) had a median cycle of 36 days or more, compared with between 7.5% and 9.6% for the lower BMI categories.

**Table 2:** Demographic and lifestyle characteristics across median cycle length.

Characteristics		Short cycle (≤20 days), n (%)	Normal cycle (21-35 days), n (%)	Long cycle (≥36 days), n (%)
**Age (years)^a^**				
	18-24	1255 (48.01)	556,541 (39.57)	80,887 (47.42)
	25-29	634 (24.25)	334,817 (23.80)	47,728 (27.98)
	30-34	330 (12.62)	248,280 (17.65)	26,429 (15.50)
	35-39	170 (6.50)	151,867 (10.80)	10,119 (5.93)
	40-55	225 (8.61)	115,138 (8.19)	5399 (3.17)
**BMI (kg/m^2^)^b^**				
	≤18.4	31 (8.1)	29,309 (9.21)	3855 (10.16)
	18.5-24.9	182 (47.6)	182,511 (57.35)	19,727 (51.98)
	25.0-29.9	85 (22.3)	63,183 (19.85)	7439 (19.60)
	30.0-34.9	40 (10.5)	26,253 (8.25)	3851 (10.15)
	35.0-50.0	42 (11.0)	16,515 (5.19)	2983 (7.86)
	≥50.0	2 (0.5)	494 (0.16)	96 (0.25)
**Menstrual flow^c^**				
	Light	240 (50.4)	37,469 (21.78)	6462 (25.77)
	Medium	222 (46.6)	130,178 (75.67)	17,836 (71.12)
	Heavy	14 (2.94)	4378 (2.54)	780 (3.11)
**Smoking status^d^**				
	Smoke regularly	160 (14.6)	94,649 (14.18)	9803 (12.56)
	Smoke sometimes	155 (14.1)	78,205 (11.72)	9146 (11.72)
	Do not smoke	782 (71.3)	494,381 (74.09)	59,115 (75.73)
**How often do you drink alcohol?^e^**				
	≥3 times a week	60 (5.5)	38,455 (5.79)	3600 (4.64)
	1-2 times a week	213 (19.5)	130,651 (19.68)	13,399 (17.25)
	<2-3 times a month	444 (40.7)	277,731 (41.84)	32,422 (41.75)
	None	373 (34.2)	217,031 (32.69)	28,236 (36.36)
**Frequency of high stress^f^**				
	≥3 times a week	448 (40.1)	224,951 (32.75)	27,495 (34.13)
	1-2 times a week	359 (32.1)	221,845 (32.30)	26,426 (32.80)
	<3 times a month	235 (21.0)	196,573 (28.62)	21,682 (26.92)
	None	75 (6.7)	43,528 (6.34)	4954 (6.15)
**Frequency of physical activity^g^**				
	≥3 times a week	191 (16.5)	127,088 (17.76)	13,008 (15.49)
	1-2 times a week	190 (16.4)	158,190 (22.10)	17,744 (21.13)
	<3 times a month	155 (13.4)	107,620 (15.04)	13,370 (15.92)
	None	622 (53.7)	322,839 (45.11)	39,853 (47.46)
**Trying to conceive^h^**				
	<1 month	63 (27.8)	32,361 (26.82)	4086 (23.93)
	1-3 months	49 (1.6)	23,828 (19.75)	3506 (20.53)
	3-6 months	35 (15.4)	14,902 (12.35)	2171 (12.71)
	>6 months to 1 year	18 (7.9)	13,096 (10.85)	1974 (11.56)
	>1 year	62 (27.3)	36,484 (30.23)	5338 (31.26)
**Number of children^i^**				
	0	650 (63.22)	444,711 (70.14)	56,615 (76.55)
	1 or 2	324 (31.52)	162,663 (25.65)	15,316 (20.71)
	≥3	54 (5.25)	26,679 (4.21)	2025 (2.74)
**Any reproductive disorders?^j^**				
	Yes	164 (16.7)	64,376 (10.47)	12,182 (17.01)
	No	563 (57.2)	403,419 (65.63)	39,615 (55.32)
	I don't know	257 (26.1)	146,896 (23.90)	19,818 (27.67)

^a^Short cycle (N=2614), normal cycle (N=1,406,643), and long cycle (N=170,562).

^b^Short cycle (N=382), normal cycle (N=318,265), and long cycle (N=37,951).

^c^Short cycle (N=476), normal cycle (N=172,025), and long cycle (N=25,078).

^d^Short cycle (N=1097), normal cycle (N=667,235), and long cycle (N=78,064).

^e^Short cycle (N=1090), normal cycle (N=663,868), and long cycle (N=77,657).

^f^Short cycle (N=1117), normal cycle (N=686,897), and long cycle (N=80,557).

^g^Short cycle (N=1158), normal cycle (N=715,737), and long cycle (N=83,975).

^h^Short cycle (N=227), normal cycle (N=120,671), and long cycle (N=17,075).

^i^Short cycle (N=1028), normal cycle (N=634,053), and long cycle (N=73,956).

^j^Short cycle (N=984), normal cycle (N=614,691), and long cycle (N=71,615).

### Cycle Length Variability

**Figure 3:**
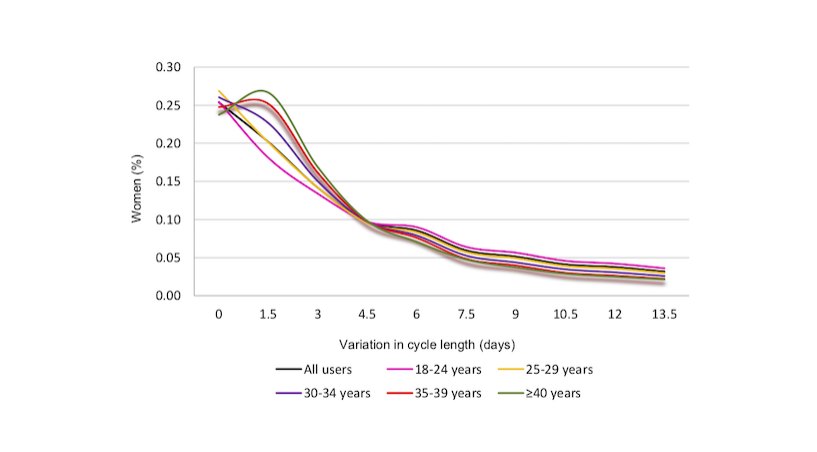
Variation in cycle length across age group.

**Figure 4:**
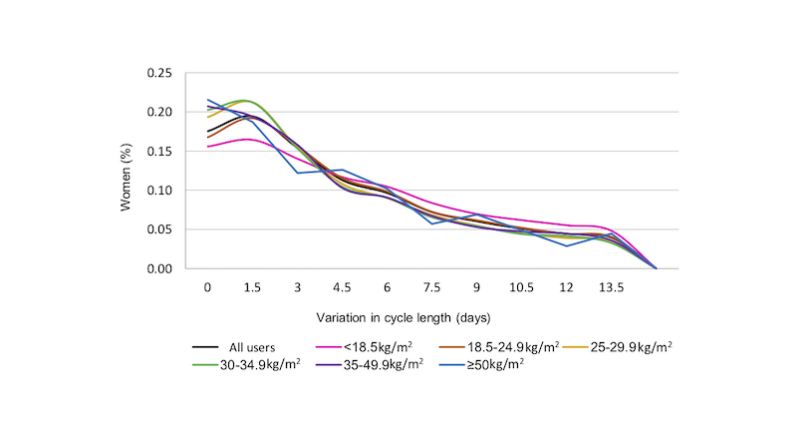
Variation in cycle length across BMI categories.

Figure 3 illustrates the variation in cycle length for all women and for each age group. Among all women, 25.37% (275,715/1,086,923) had a cycle length variation of 0 to 1.5 days and 69% (753,831/1,086,923) of women had a <6-day variation. Variation in cycle length of 1.5 to 4.5 days was more common for women aged ≥35 years than for women aged <29 years, and this was particularly prominent at 1.5 days variation. That is, in women aged 35 to 39 years, and ≥40 years, a 1.5-day variation in cycle length was found in 25.17% (27,754/110,252) and 26.64% (14,255/53,502) of women, respectively, compared with 18.08% (63,314/350,021) and 20.12% (45,683/227,109) of women aged between 18 and 24 years and 25 and 29 years, respectively. Normal cycle variation between 3 and 4.5 days was also higher in women aged between 35 and 39 years (16.11%; 17,761/110,252) and ≥40 years (16.85%; 9015/53,502) compared with women aged between 18 and 24 years (13.38%; 46,817/350,021) and 25 and 29 years (14.17%; 32,185/227,109). Comparatively, a variation of ≥6 days was more prominent in the younger women and tracked similarly with increasing age. Across BMI groups, women with a higher BMI tended to have less variation in their cycle. This was particularly evident with a cycle variation of ≥4.5 days, where there were fewer women with a higher BMI and more women with a low BMI (<18 kg/m^2^; Figure 4).

### Ovulation and Phases of the Menstrual Cycle

There were 18,761 cycles with positive LH ovulation tests, signifying the end of the follicular phase, and the following day to be the day of estimated ovulation. The highest percentage of cycles was at an estimated ovulation day 14 (13.08%, 2509 cycles; Table 3), indicating a 13-day follicular phase (Multimedia Appendix 1). There was a similar percentage of cycles with estimated ovulation at day 13 (2248 cycles, 12.13%) and day 15 (2267 cycles, 11.96%). The number of cycles with estimated ovulation continued to reduce outside these days. Across age groups (Multimedia Appendix 2), the highest percentage of cycles was at an estimated ovulation day 15 for women aged between 18 and 24 years (187/1687 cycles, 11.08%), day 14 for women aged between 25 and 39 years (2108 cycles, 12%-15%), but at day 12 for women aged 40 years (237/1594 cycles, 14.9%). Across BMI categories (Multimedia Appendix 3), more frequent positive ovulation tests occurred at day 14, indicating a 13-day follicular length, except for a BMI of 35 to 49.9 kg/m^2^, which was highest at day 13 (80 cycles).

The length of the luteal phase was highest at day 15, with 16.96% (3696/21,788) of the cycles, followed by a 14-day luteal phase, with 16.17% (3523/21,788) of the cycles (Table 4). The number of cycles reduced to around 12% at a 13-day and 16-day luteal phase length and dropped to 6% to 7% at a 12-day and 17-day luteal phase length. The luteal phase length across age and BMI are reported in Multimedia Appendices 4 and 5, respectively. For all age groups, the number of cycles was highest for a 15-day luteal phase length, followed by a 14-day luteal phase length. There was a higher percentage of cycles with shorter (5 to 10 day) luteal phases among the youngest compared with the higher age groups, but for a luteal phase of 13 to 16 days, more cycles were present in the age group of 25 years. Comparatively, there was a higher percentage of cycles from women aged ≥40 years that had longer luteal phases (≥15 days; Multimedia Appendix 4). A luteal phase length of 15 days was most common across all BMI categories except for the 30.0 to 34.9 kg/m^2^ category, which was at a length of 14 days. Overall, the percentage of cycles tended to track similarly across BMI categories; however, there tended to be a higher percentage of cycles with a luteal phase of 11 to 13 days from overweight (25.0 to 29.9 kg/m^2^) and obese (30.0 to 50.0 kg/m^2^) women compared with the other BMI categories (Multimedia Appendix 5).

**Table 3:** Estimated ovulation day based on positive luteinizing hormone test (N=18,761 cycles).

Estimated day of ovulation	Cycles, n (%)
11	1668 (8.89)
12	1877 (10.00)
13	2248 (11.98)
14	2509 (13.37)
15	2267 (12.08)
16	1905 (10.15)
17	1518 (8.09)
18	1154 (6.15)
19	822 (4.38)
20	636 (3.39)
21	458 (2.44)
22	321 (1.71)
23	304 (1.62)
24	266 (1.41)
25	189 (1.00)
26	184 (0.98)
27	149 (0.79)
28	160 (0.85)
29	126 (0.67)

**Table 4:** Estimated luteal phase length based on positive luteinizing hormone test (N=18,761).

Length of luteal phase (days)	Cycles, n (%)
5	142 (0.75)
6	172 (0.91)
7	206 (1.09)
8	227 (1.20)
9	344 (1.83)
10	543 (2.89)
11	946 (5.04)
12	1565 (8.34)
13	2645 (14.09)
14	3523 (18.77)
15	3696 (19.70)
16	2765 (14.73)
17	1819 (9.69)
18	1380 (7.35)
19	1006 (5.36)
20	809 (4.31)

## Discussion

### Principal Findings

We have reported recent and comprehensive data characterizing over 1.5 million women and their menstrual cycle patterns. Approximately two-thirds of women using the app were aged <30 years and one-third were overweight or obese based on their BMI. The majority of women (1,439,613/1,579,819, 1.13%) had a usual median cycle length of 21 to 35 days, of whom 16.32% (257,889/1,579,819) had a median cycle length of 28 days. Shorter cycles with less variation were more common for women ≥40 years. Among women who reported short cycles, a higher percentage reported higher stress levels, no physical activity, and a lighter menstrual flow in comparison with women with normal or long cycles. There were only subtle differences in length and variability across BMIs; however, women with the lowest BMI tended to have the highest variation in cycle length and more women with a BMI >35 kg/m^2^ had a median cycle of 36 days and longer.

### Strengths and Limitations

The reporting of menstrual cycle length and patterns was estimated from mobile phone data collected from women using the app. Although this app has a global reach of 100 million women using the app, and 30 million active users monthly, not all women reported on their BMI or other lifestyle characteristics. This means these findings cannot be generalized to all women using the app. Recently, a review of pregnancy apps and their use in culturally and linguistically diverse women found that engagement with apps may be lower in areas of higher social disadvantage, cultural and language barriers, and health literacy [36]. This study did not ask about reasons for using the app (except if it was to become pregnant); thus, it is unclear whether women who provided more demographic and fertility details have a different level of knowledge or interest in reproductive health. We did not have data to assess cultural disparities across menstrual cycle characteristics, which would be valuable in future research. Information on cycle intensity was self-reported based on subjective feelings of *light, medium,* and *heavy* flow; objective measures of menstrual flow would improve the accuracy of cycle intensity. We used the data on women to assess menstrual cycle characteristics, but also used the number of cycles to assess ovulation. Understanding the characteristics of women that contribute to anovulatory cycles would provide important information on other potential issues with fertility. Among women who have reproductive health issues, including polycystic ovary syndrome or endometriosis, the ability to become pregnant may already be reduced. An app estimating ovulation or suggesting the most fertile period for those women may be limited in its effectiveness.

The strengths of this study include the very large cohort of women providing fertility information, along with data on a high number of ovulatory cycles. The Flo app uses artificial intelligence–based algorithms, and when additional data, such as basal body temperature and ovulation data, are entered, the predictive ability improves. Comparatively, calendar-based apps may only use simulated 28-day cycles to estimate ovulation, or predictive methods may be used; for example, the standard days method, where users avoid unprotected intercourse during cycle days 8 through 19 [37] or the rhythm method, which predicts fertile days using a formula and is based on data from the menstrual records of the past 6 cycles [6]. They do not provide information on how the predictions are calculated or the accuracy of predictions.

### Comparison With Prior Work

Imperative to our study is that we assessed *individual* women, and we report on the number and percentage of women and median menstrual cycle length. Our results are useful in the clinic setting and provide information on the actual occurrence of cycle data in women but also differences in cycle patterns across age groups, BMI, and some lifestyle characteristics. Importantly, we show that a cycle length of 28 days is not common for many women, which is usually the reference length for a typical woman. Comparatively, previous analyses among women using fertility apps or cohort studies have reported that the mean or median cycle length for all women ranged between 28 and 30 days [8,11,33,34], and studies reporting on actual cycles reported means between 27.7 and 29.6 days [2,4,5,7,35]. These results cannot be extrapolated to an individual woman, and clinically, it is not helpful, as we have clearly demonstrated that the percentage of women with an average cycle length of 28 days is uncommon. Our results provoke a potential paradigm shift, changing our thinking from the current concept of mean cycle length to frequencies of cycle length in individual women.

Our results are consistent with previous studies reporting on shorter cycle lengths with increasing age [3-5,11], but also less variation with older age [5,13]. Additionally, there is a dependence between age and length of the luteal phase. Only a small number of cycles were assessed in our luteal phase data, but our results are partly consistent with the study by Lenton et al [12] showing a higher incidence of shorter cycles in the youngest women (18-24 years) but also the oldest women aged between 45 and 50 years. A recent study by Bull et al [4] using a fertility app did not show any difference in the luteal phase across age groups. Such differences are likely because of the assessment of this phase, and when using an app, one that includes thermal changes, but also objective markers of ovulation are more likely to provide accurate information.

Epidemiological evidence has demonstrated the adverse effects of obesity on female reproduction, including anovulation and menstrual cycle irregularities [24,25,38], impaired pregnancy success using assisted reproductive technology, and infertility [39]. Furthermore, mechanistic studies have confirmed alterations in reproductive hormones associated with obesity [40]. Small studies demonstrated body composition measures associated with menstrual cycle irregularities [23-26], and an Australian study of 726 women was the first to show that obese women were twice as likely to have greater cycle variation (>15 days) compared with women with a normal weight [9]. In a recent app study using >600,000 cycles, there were no differences in the percentage of cycles across BMI categories, except morbidly obese women had higher cycle length variation by 0.4 days and longer follicular phase length by 0.9 days, than women with a normal BMI [4]. The current analysis revealed that median menstrual cycle length and length of follicular and luteal phases were not remarkably different with increasing BMI, except for the heaviest women at a BMI of ≥50 kg/m^2^. It is interesting to note that of the over 1.5 million women in the study, only 22.57% (356,598/1,579,819) reported height and weight to calculate BMI. Although this might demonstrate some information bias such that our findings may systematically underestimate the relationship between BMI and menstrual cycle, the proportion of women who were overweight or obese (33.93%) in this study is similar to the current prevalence rates in the general and reproductive population of women [27,41]. Thus, the effect of BMI on menstrual cycle length may be mediated by other factors that we did not assess in our descriptive study. Given the inconsistent findings and lack of clear association between BMI and menstrual cycle characteristics in large cohorts of women (rather than data on cycles), further investigation into this area is needed.

This is the largest analysis to report on physical activity, stress, and menstrual flow across short, normal, and long menstrual cycle lengths. Interestingly, shorter cycles were associated with a higher frequency of high stress, no physical activity, and lighter menstrual flow. There is limited information on stress and physical activity, but research to date suggests that professional and high-frequency training impairs ovarian activity, which may manifest as luteal phase defects, irregular menstruation, or amenorrhea [42-44]. In nontrained women aged 18 to 44 years, total recreational physical activity and vigorous recreational activity were positively associated with cycle length, but not cycle variability [45]. Regarding sedentary behavior, among 2613 nulliparous Danish women, those who were sedentary (ie, <5 metabolic equivalents [METs] per week of physical activity), had a higher prevalence of irregular cycles than women who engaged in moderate levels of activity (20 to 39 METs per week; probability ratio 1.54; 95% CI 1.16-2.04) [46]. Small cross-sectional studies have shown that higher stress is a significant predictor of irregular menstrual cycles [47] and dysmenorrhea [48]. Although these findings support the benefits of moderate physical activity and minimizing stress, further investigation into the impact of lifestyle factors on menstrual cycle characteristics is necessary.

The current findings have clinical implications for women who are trying to conceive but also for women with different lifestyle characteristics. First, current estimations for conceiving are based on the premise of a 28-day cycle with ovulation on day 14. We demonstrated that only 16% of women had a median 28-day cycle. Our results invoke a fundamental change in the assessment of cycle lengths of individual women and that the population mean cycle length may no longer be an applicable form of its measurement. Second, few menstrual cycle differences were seen in women with different BMIs. Finally, lifestyle factors such as physical activity and stress may relate to cycle length and therefore the potential day of ovulation. Prediction models, including menstrual cycle data as well as age and lifestyle factors, could be developed to predict the likelihood of pregnancy success. The use of such models would be helpful in the clinic setting to optimize conception, particularly in those who do not have regular cycles.

### Conclusions

On a global scale, we have provided extensive evidence on the characteristics of women and their menstrual cycle length and patterns. We demonstrate that the typical average 28-day cycle length is not common for a high percentage of women, and only 13.08% of cycles had an estimated ovulation on day 14. Age appeared to be a more important factor than BMI in terms of menstrual cycle length and variability. Our data provide important information on the necessity for an individualized approach to support reproductive health and fertility, and modifiable factors such as physical activity and stress should additionally be considered when planning a pregnancy. Future work could extend these findings by addressing cultural and ethnic diversities in relation to menstrual cycle patterns.

## Appendix

Multimedia Appendix 1 Estimated length of the follicular phase.

Multimedia Appendix 2 Estimated ovulation day based on positive luteinizing hormone test across age groups (N=18,761 cycles).

Multimedia Appendix 3 Estimated ovulation day based on positive luteinizing hormone test across BMI group (N=18,235 cycles).

Multimedia Appendix 4 Number of cycles and the luteal length, according to age.

Multimedia Appendix 5 Number of cycles and the luteal length, according to BMI.

## Abbreviations

H: heavy
L: light
LH: luteinizing hormone
M: medium
MET: metabolic equivalent
