# Supplementary figures and images for: Menstrual Cycle Length and Patterns in a Global Cohort of Women Using a Mobile Phone App: Retrospective Cohort Study
Source: J Med Internet Res. 2020 Jun 24;22(6):e17109. doi: 10.2196/17109 (PMC7381001; doi:10.2196/17109)

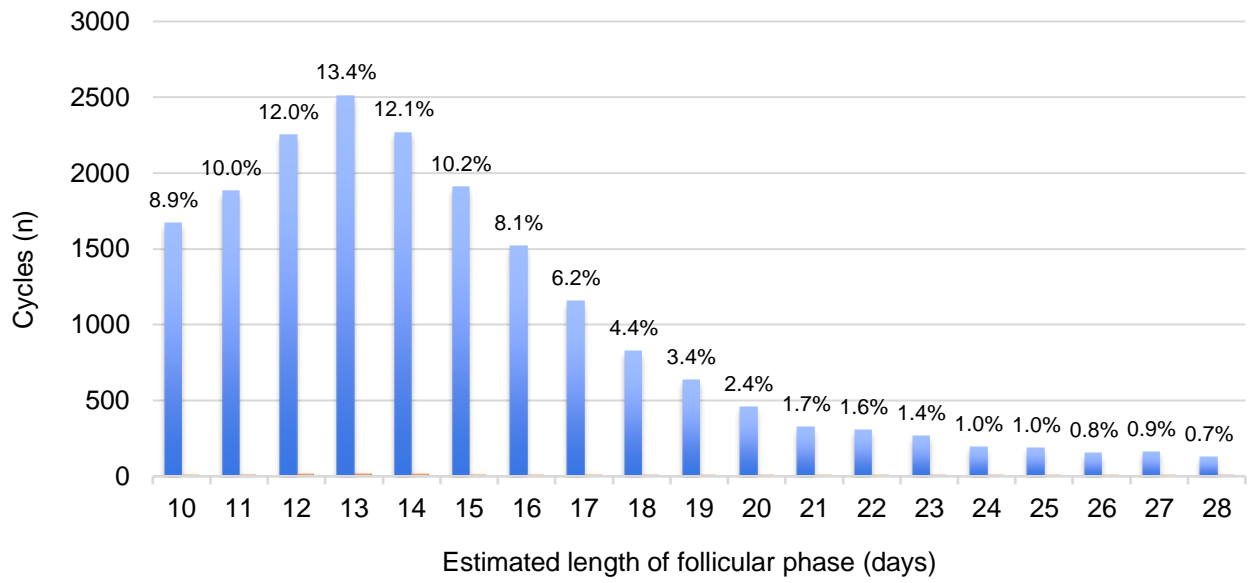

Supplement: Multimedia Appendix 1 [file jmir_v22i6e17109_app1.pdf]

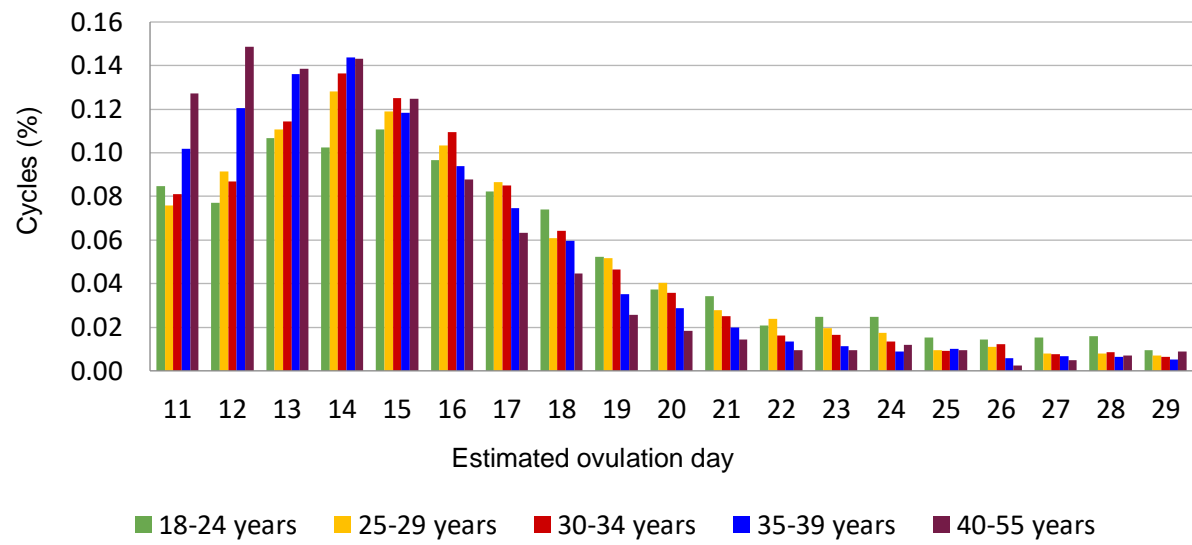

Supplement: Multimedia Appendix 2 [file jmir_v22i6e17109_app2.pdf]

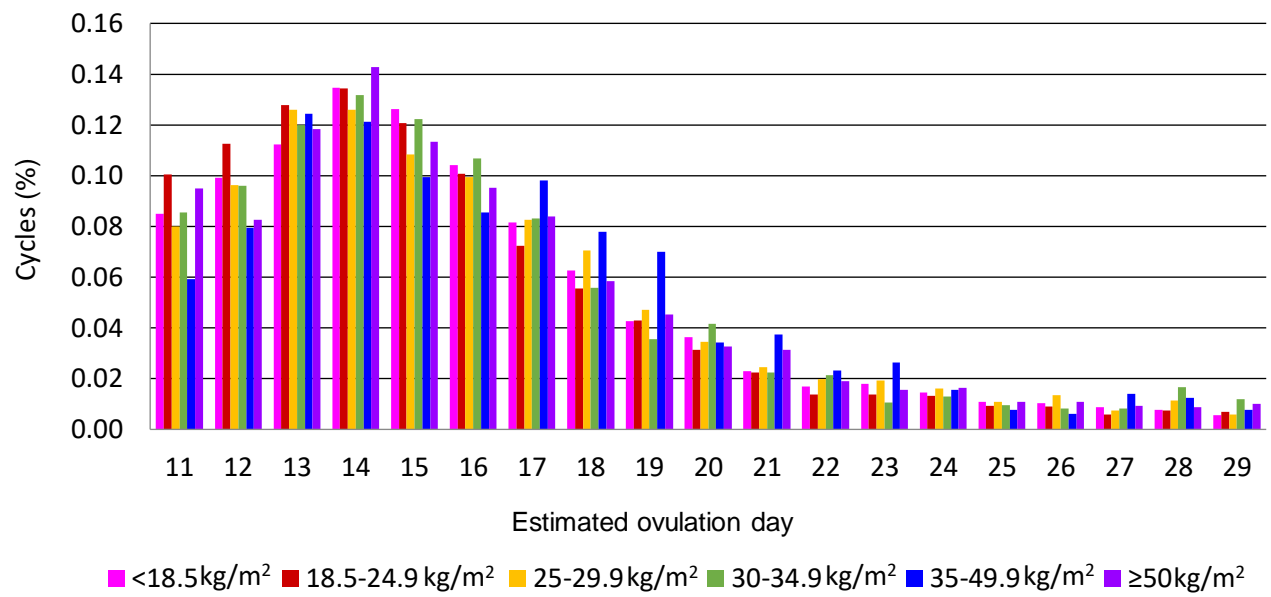

Supplement: Multimedia Appendix 3 [file jmir_v22i6e17109_app3.pdf]

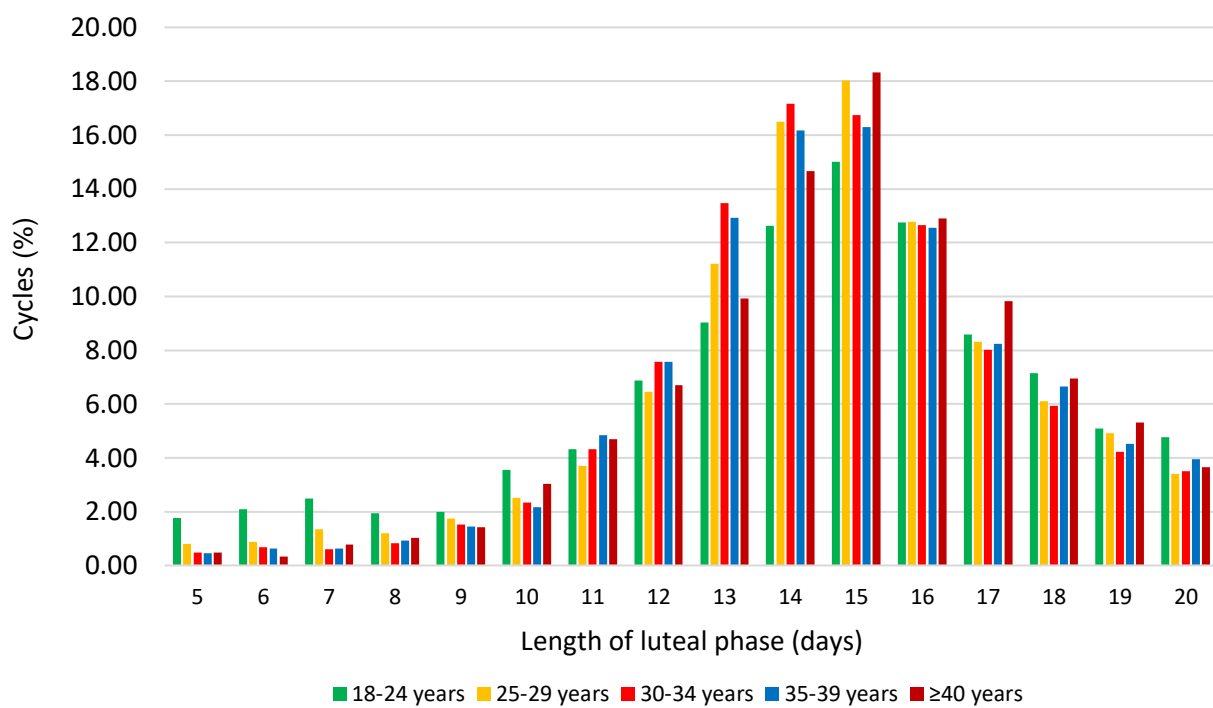

**Multimedia file 5:** Number of cycles and the luteal length, according to BMI.

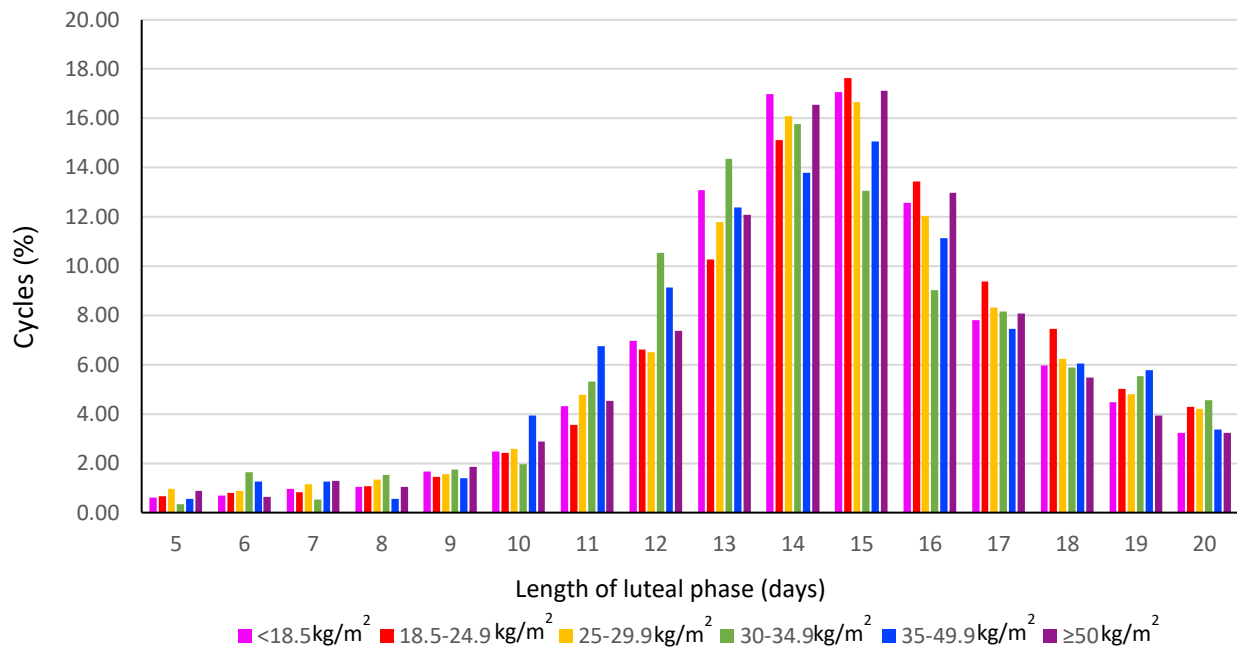

Supplement: Multimedia Appendix 4 [file jmir_v22i6e17109_app4.pdf]

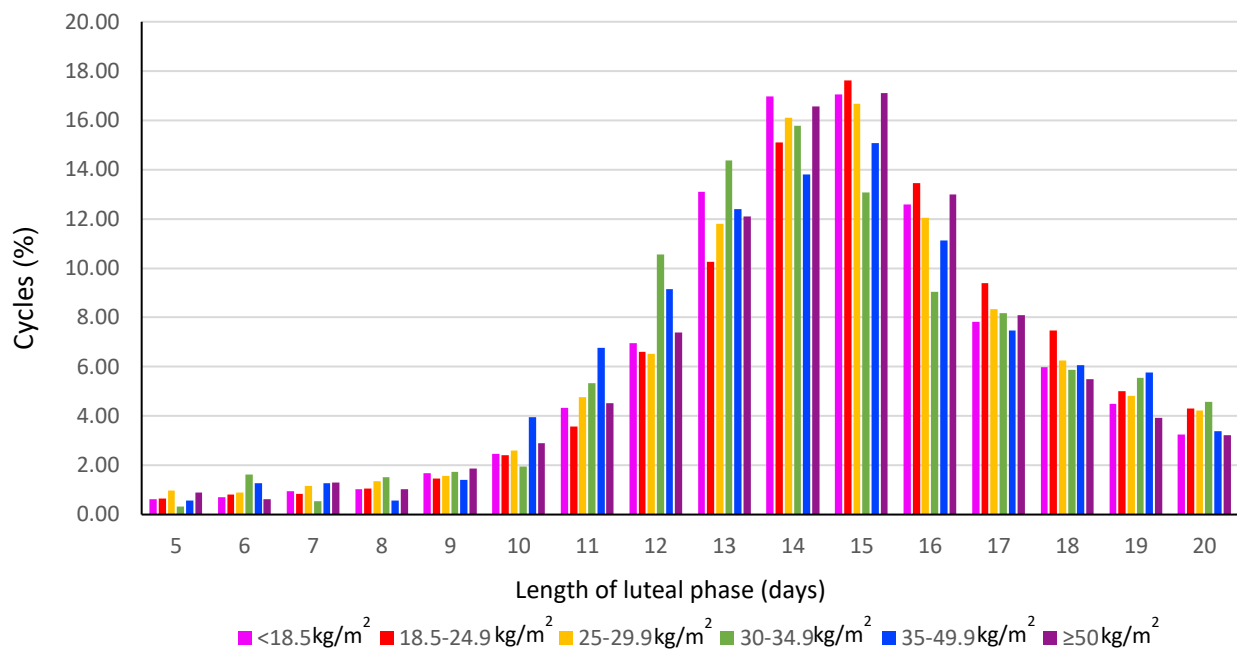

Supplement: Multimedia Appendix 5 [file jmir_v22i6e17109_app5.pdf]
